# YycH and YycI Regulate Expression of *Staphylococcus aureus* Autolysins by Activation of WalRK Phosphorylation

**DOI:** 10.3390/microorganisms8060870

**Published:** 2020-06-09

**Authors:** Mike Gajdiss, Ian R. Monk, Ute Bertsche, Janina Kienemund, Tanja Funk, Alina Dietrich, Michael Hort, Esther Sib, Timothy P. Stinear, Gabriele Bierbaum

**Affiliations:** 1 Institute of Medical Microbiology, Immunology and Parasitology, University Hospital Bonn, 53125 Bonn, Germany; m.gajdiss@uni-bonn.de (M.G.); Jkienemund@web.de (J.K.); tanja@tfunk.de (T.F.); dietrich.alina@gmx.de (A.D.); s6mihort@uni-bonn.de (M.H.); Esther.Sib@ukbonn.de (E.S.); 2 Department of Microbiology and Immunology, Doherty Institute for Infection and Immunity, University of Melbourne, Melbourne, VIC 3010, Australia; ian.monk@unimelb.edu.au (I.R.M.); tstinear@unimelb.edu.au (T.P.S.); 3 Department of Infection Biology, University of Tuebingen, 72076 Tuebingen, Germany; bertsche.ute@uni-hohenheim.de

**Keywords:** WalK, WalR, two-component regulatory system, *Staphylococcus aureus*, YycH, YycI, signal

## Abstract

*Staphylococcus aureus* is a facultative pathogen that can encode numerous antibiotic resistance and immune evasion genes and can cause severe infections. Reduced susceptibility to last resort antibiotics such as vancomycin and daptomycin is often associated with mutations in *walRK*, an essential two-component regulatory system (TCS). This study focuses on the WalK accessory membrane proteins YycH and YycI and their influence on WalRK phosphorylation. Depletion of YycH and YycI by antisense RNA caused an impaired autolysis, indicating a positive regulatory function on WalK as has been previously described. Phosphorylation assays with full-length recombinant proteins in phospholipid liposomes showed that YycH and YycI stimulate WalK activity and that both regulatory proteins are needed for full activation of the WalK kinase. This was validated in vivo through examining the phosphorylation status of WalR using Phos-tag SDS-PAGE with a *yycHI* deletion mutant exhibiting reduced levels of phosphorylated WalR. In the *yycHI* knockdown strain, muropeptide composition of the cell wall was not affected, however, the wall teichoic acid content was increased. In conclusion, a direct modulation of WalRK phosphorylation activity by the accessory proteins YycH and YycI is reported both in vitro and in vivo. Taken together, our results show that YycH and YycI are important in the direct regulation of WalRK-dependent cell wall metabolism.

## 1. Introduction

In bacteria, the adaptation to environmental conditions and sensing of the metabolic status of the cell is often mediated by two-component regulatory systems (TCS). The core genome of *Staphylococcus aureus*, a commensal pathogen that is infamous for causing severe nosocomial and community acquired infections, harbors 16 sets of TCS, of which 15 are not essential for bacterial growth [1]. WalRK, however, is essential to maintain cell wall metabolism, mainly by controlling the transcription of cell wall lysing enzymes that are essential for growth [2,3,4]. First discovered in *Bacillus subtilis* [5] and later in *S. aureus* [6], this TCS comprises the membrane-bound histidine kinase WalK and the cytoplasmic response regulator WalR. The genes for two additional membrane proteins, *yycH* and *yycI*, are co-transcribed with *walR* and *walK* [7]. An additional cytoplasmic protein encoded by *yycJ*, a gene located downstream of the *walRK* operon is controlled by a separate promoter and does not show any functional relation to the WalRK TCS in *S. aureus* [7].

In addition to its role in cell wall metabolism, WalRK has been reported to be involved in *S. aureus* host colonization [8], virulence [2,4], and biofilm formation [3]. The highest activity of the WalRK TCS occurs at the end of the log phase in *B. subtilis* [5] and in *S. aureus* [9] and entails autophosphorylation of the membrane bound WalK kinase, followed by phosphotransfer to the cytoplasmic response regulator WalR. Activation by phosphorylation leads to dimerization and binding to specific promoter regions, thereby altering the transcription level of the corresponding genes. Among the WalRK-controlled genes in *S. aureus*, several code for peptidoglycan hydrolases, including the muramidases IsaA and SceD, the endopeptidase LytM, the CHAP domain autolysins SsaA, SA0620, SA0710, SA2097, and SA2353, the amidase Sle1, as well as AtlA, the major autolysin, which has a glucosaminidase and an amidase activity [3].

Several studies have highlighted the role of WalR, WalK, YycH, and YycI in resistance of *S. aureus* to the last resort antibiotic vancomycin. During vancomycin therapy of MRSA in nosocomial infections, changes in expression of the WalRK TCS [10], and/or amino acid exchanges in WalK or WalR have often been reported to convert vancomycin susceptible *S. aureus* to homogeneous vancomycin-intermediate *S. aureus* (VISA) or heterogeneous VISA (hVISA) [11,12,13]. The main mechanism of non-susceptibility is preventing the antibiotic to reach its target molecule, the d-alanyl-d-alanine moiety of the ultimate peptidoglycan precursor molecule lipid II in the cytoplasmic membrane. This is achieved through cell wall thickening and reduced crosslinking of the peptidoglycan, leading to a higher abundance of free d-alanyl-d-alanine residues, which causes vancomycin to be bound in the peripheral cell wall [14,15].

**Figure 1:**
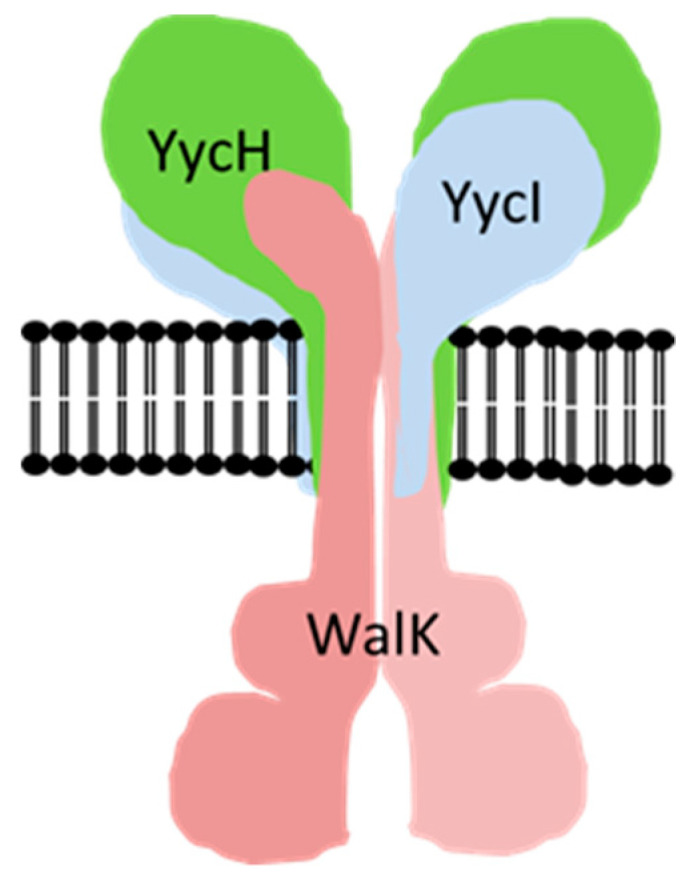
Schematic drawing of the complex formed by the WalK dimer and the accessory proteins YycH and YycI in the cytoplasmic membrane as proposed by Fukushima et al. (2008) [23] for *B. subtilis*.

In *B. subtilis*, the two accessory proteins YycH and YycI were reported to negatively control WalRK activity [16]. Here, YycH and YycI are membrane proteins with large extracellular domains (YycH, 427 amino acids; YycI, 250 amino acids) and very small N-terminal intracellular domains (YycH, 18 amino acids; YycI, 8 amino acids) (Figure 1). The crystal structures of the *B. subtilis* YycH and YycI have been solved and both proteins share a common fold [17,18]. In non-dividing *Bacillus* cells, WalK, YycH, and YycI are distributed across the cell membrane, allowing the formation of a complex. Cells lacking YycH showed a stronger transcription of autolysins [19], indicating that the formation of the complex leads to a reduction of WalK activity [16,20]. A hexameric model, comprising two YycH, two YycI, and one dimer of WalK was proposed for the complex by in silico modeling of the membrane domains and supported by mutagenesis studies [21] (Figure 1). A strong complex formation and inhibition of WalK activity was seen only, when both proteins, YycH and YycI, were present [16,20]. During septum formation, WalK is located at the division septum where no inhibitory complex with YycH and YycI is formed and, therefore, the presence of the cell wall biosynthetic complex was proposed to serve as signal for WalK activity [22]. However, recently, evidence was presented, that a signaling activity of WalK in *B. subtilis* is still possible in the absence of the septal cell wall biosynthetic complex [20]. Furthermore, it was demonstrated, that the extracellular domains of YycH and YycI are not involved in signaling in *B. subtilis* and that yet unidentified cell wall fragments produced by the D,L-endopeptidases LytE and CwlO are able to modify the activity of WalK and therefore probably act as signals for WalK [20].

Much less is known about the role of YycH and YycI in *S. aureus*. As in *B. subtilis*, both proteins are membrane proteins with small N-terminal domains and large extracellular domains. In contrast to the results obtained with *B. subtilis*, fluorescence microscopy of GFP-tagged proteins revealed a localization of YycH together with WalK to the septal region in exponential growth phase [7]. The disruption of the *yycH* and *yycI* genes led to a downregulation of the WalRK regulon, including the expression of the autolysin genes *atlA* and *sle1* [13]. In these mutants, a reduced Triton X-100 induced autolysis indicated an activating regulatory function of the two accessory proteins on the WalRK TCS, which is opposite to the role of the proteins in *B. subtilis* [22]. The presence of both proteins YycH and YycI together with WalK was necessary for a high expression of the WalRK controlled genes and both proteins were necessary for interaction with WalK in a bacterial-two hybrid system, most probably forming a ternary complex via their transmembrane domains [13]. Mutations in YycH and YycI that led to N-terminal truncations of YycH [13,24] or YycI [22] resulted in a reduced expression of autolysin genes and a phenotype with reduced susceptibility to vancomycin [13]. Nevertheless, direct evidence for YycH and YycI impacting on WalK autophosphorylation activity or the phosphorylation of WalR has not been reported for *B. subtilis* or *S. aureus* so far.

In this study, we present in vitro and in vivo analysis of WalRK phosphorylation activity in *S. aureus* to investigate the regulatory role of the accessory proteins YycH and YycI. For in vitro analysis, the full-length proteins were purified and reconstituted into phospholipid liposomes in various combinations and phosphorylation activity was tested. Additionally, a reduction of *yycH* and *yycI* gene expression was achieved by antisense RNA, which led to a reduced autolysis and a higher wall teichoic acid (WTA) content of the cell wall. Visualizing the phosphorylation state of WalR in cell lysates, we observed an impaired phosphorylation of WalR in a Δ*yycHI S. aureus* strain in comparison to the wild-type, which underlines the activating regulatory function of the YycH and YycI proteins. We tested several cell wall components for activation or inhibition of the WalK kinase in vitro but did not observe any impact on the phosphorylation of WalK and WalR.

## 2. Materials and Methods

### 2.1. Creation of yycH, yycI, and yycHI Antisense Knockdown Clones and yycHI Deletion

**Figure 2:**
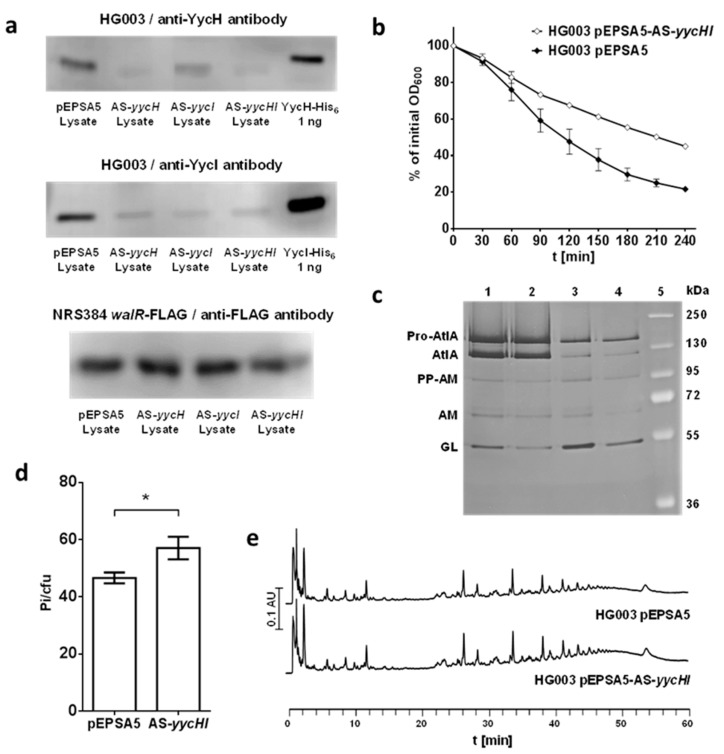
Characterization of *S. aureus* HG003 with antisense knockdown of the YycH and YycI proteins. (**a**) Western blots of *S. aureus* HG003 and *S. aureus* NRS384 *walR*-FLAG lysates with the pEPSA5, pEPSA5-AS-*yycH*, pEPSA5-AS-*yycI*, and pEPSA5-AS-*yycHI* vectors as well as recombinant YycH-His6 and YycI-His6. Antisense RNA against *yycHI* reduced the translation of both proteins. Knockdown of *yycH* reduced the abundance of YycI and vice versa. Expression of the same antisense plasmids in *S. aureus* NRS384 *walR*-FLAG showed no significant reduction of WalR abundance. (**b**) Triton X-100 induced autolysis is reduced in the *S. aureus* HG003 *yycHI* knockdown strain. Cells of *S. aureus* strains HG003 pEPSA5 and HG003 pEPSA5-AS-*yycHI* were harvested in late-log phase, washed, and resuspended in TBS containing 0.1% Triton X-100. Lysis was observed by OD_600_ measurements every 30 min. Error bars indicate standard deviation of two independent experiments. Similar results were obtained with *S. aureus* RN4220 pEPSA5 and RN4220 pEPSA5-AS-*yycHI* (data not shown). (**c**) Zymogram of LiCl autolysin extracts from *S. aureus* strains NRS384 *walR*-FLAG (1), NRS384 *walR*-FLAG *ΔyycHI* (2), HG003 pEPSA5 (3), HG003 pEPSA5-AS-*yycHI* (4). (5): PageRuler Plus Prestained Protein Ladder (Thermo Fisher Scientific, Schwerte, Germany). Pro-AtlA (138 kDa): Full-length AtlA with propeptide; AtlA (113 kDa): Full-length AtlA without propeptide; PP-AM (87 kDa): Amidase domain with propeptide; AM (62 kDa): Amidase domain; GL (51 kDa): Glucosaminidase domain; Designations according to [28]. For better visibility, the image of the methylene blue stained gel was converted to greyscale and inverted. The antisense knockdown of *yycHI* in HG003 as well as deletion of the *yycHI* genes in NRS384 resulted in decreased lysis of *M. luteus* in the zymogram; (**d**) Inhibition of *yycHI* transcription increases WTA content of the cell wall. Cell walls of *S. aureus* strains HG003 pEPSA5 and HG003 pEPSA5-AS-*yycHI* were purified and WTA content was compared by determination of the inorganic phosphate content. Error bars indicate standard deviation of three independent experiments. *p* value of two-tailed t-test was 0.0149 and considered significantly different (*p* < 0.05). (**e**) UPLC analysis of mutanolysin-digested peptidoglycan of *S. aureus* HG003 pEPSA5 and HG003 pEPSA5-AS-*yycHI*. Induction of the *yycHI* antisense knockdown did not cause changes in muropeptide composition and cross-linking.

Primers AS-*yycH*_for, AS-*yycH*_rev, AS-*yycI*_for, AS-*yycI*_rev, AS-*yycHI*_for, and AS-*yycHI*_rev (Table 1) were used to create antisense inserts for the *yycH* (223 bp) and *yycI* (189 bp) genes as well as a region overlapping the last 67 bp of *yycH* and the first 100 bp of *yycI* for the inhibition of transcription of both genes. The PCR products were digested with XbaI and EcoRI and ligated into the pEPSA5 vector [25] in antisense direction, resulting in the antisense plasmids pEPSA5-AS-*yycH*, pEPSA5-AS-*yycI*, and pEPSA5-AS-*yycHI*. The constructs were first transformed into CaCl_2_-competent *Escherichia coli* JM109 cells, then electroporated into *S. aureus* RN4220 and finally into *S. aureus* HG003 cells, which is a derivative of the widely used model strain *S. aureus* NCTC 8325, in which the mutations in *tcaR* and *rsbU* have been repaired [26]. Sanger sequencing confirmed all constructs. Reduction of the protein expression was controlled by Western blotting with specific anti-*YycH* and anti-YycI antibodies (Figure 2). The pIMAY-ZΔ*yycHI* deletion vector was constructed as described previously [9]. The plasmid was transformed into *S. aureus* NRS384 *walR*-FLAG with allelic exchange conducted as described by Monk et al. [27]. *S. aureus* NRS384 is a member of the USA300 community associated epidemic MRSA [9].

### 2.2. Western Blot for Detection of YycH and YycI in Cell Lysates

Fresh preparations of recombinant YycH-His_6_ and YycI-His_6_ proteins were used to immunize white New Zealand rabbits and to obtain affinity purified specific polyclonal antibodies (performed by Davids Biotechnology GmbH, Regensburg, Germany). The antibodies were used for detection of YycH and YycI proteins in cell lysates obtained from log phase *S. aureus* HG003 cells containing the plasmids pEPSA5, pEPSA5-AS-*yycH*, pEPSA5-AS-*yycI*, and pEPSA5-AS-*yycHI*. The cells were grown in TSB supplemented with 34 µg/mL chloramphenicol and 50 mM xylose to induce the expression of the antisense plasmids. Cells from late log phase were mixed with one sample volume of ice cold ethanol:acetone (1:1) and harvested by centrifugation at 7300× *g* for 5 min at 4 °C. The cells were washed with 20 mL of ultrapure water and resuspended in 500 μL of TBS (50 mM Tris-HCl, 150 mM NaCl, pH 7.5). Cells were disrupted by bead beating three times at 5000 rpm for 30 s (Precellys 24, Bertin Instruments) and then the lysates were centrifuged at 11,000× *g* for 5 min at 4 °C. The protein concentration of the cell lysates was determined (Protein Assay Dye Reagent Concentrate, Bio-Rad) and an equal amount of protein for each strain was run in a conventional 10% SDS-PAGE gel. Then, 1 ng of recombinant YycH-His_6_ or YycI-His_6_ was loaded on the same gel to compare the migration distance of the detected bands in cell lysates with the purified proteins. The gel was washed three times with TBS-T (TBS, 0.05% Tween 20, pH 7.5) and blotted to a PVDF membrane. Membranes were blocked with blocking buffer (5% Easyblocker in TBS-T) overnight. The membrane was incubated with 10 µg/mL of the primary anti-YycH and anti-YycI antibodies in blocking buffer and then with the secondary antibody (Goat anti-Rabbit IgG (H+L) Secondary Antibody HRP, Thermo Fisher Scientific) at a dilution of 1:2000 in blocking buffer. The membrane was washed three times with TBS containing 0.05% Tween 20 and bound antibody was detected using the WesternSure^®^ PREMIUM Chemiluminescent Substrate (LI-COR Biosciences, Bad Homburg, Germany) and the C-DiGit^®^ Blot Scanner (LI-COR Biosciences).

### 2.3. Detection of WalR Phosphorylation by Phos-Tag SDS-PAGE and Western Blot

Stationary phase cultures of *S. aureus* NRS384 *walR*-FLAG and *S. aureus walR*-FLAG Δ*yycHI* in TSB were diluted 1:1000 into 1000 mL of fresh TSB and growth at 37 °C was observed by measuring the OD_600_ every 30 min. Samples were taken after 120, 150, 180, and 240 min, and the extraction of total cellular protein was conducted as described above. A total of 10 μg of protein per lane was loaded on an 8% SDS-PAGE gel containing 50 μM Phos-tag acrylamide (Wako Chemicals, Neuss, Germany) and 100 μM MnCl_2_. The gel was run according to the manufacturer’s instructions (Wako Chemicals). To remove manganese ions before blotting, the gel was washed two times with transfer buffer (25 mM Tris, 192 mM glycine, 20% methanol, pH 8.3) containing 1 mM EDTA and once with transfer buffer without EDTA. The separated proteins were blotted onto a PVDF membrane using the Trans-Blot^®^ Turbo™ transfer system (Bio-Rad) according to the manufacturer’s instructions. The membrane was treated with blocking buffer (5% EasyBlocker (GeneTex) in TBS, 0.05% Tween 20) for 16 h at RT and then with blocking buffer containing 1:500 mouse anti-FLAG^®^ M2-Peroxidase (HRP) monoclonal antibody (Sigma, Taufkirchen, Germany) for 1 h at RT. Antibody detection was conducted as described above and the ratio of phosphorylated WalR to total WalR was calculated by quantification of the Western blot bands using GelAnalyzer 2010a.

### 2.4. Triton X-100 Autolysis and Light Microscopy

Strains *S. aureus* HG003 pEPSA5 and HG003 pEPSA5-AS-*yycHI* were grown with aeration in TSB containing 50 mM xylose to induce transcription of the antisense RNA and without xylose as a control. At an OD_600_ of 0.6, the cells were cooled on ice and harvested by centrifugation. The pellets were washed once with ultrapure water (Milli-Q treated deionized water), resuspended in PBS (pH 7), and autolysis was induced by the addition of 0.1% Triton X-100. The cells were incubated at 37 °C with constant shaking and lysis was observed photometrically at 600 nm every 30 min.

For light microscopy, 5 mL of TSB supplemented with chloramphenicol (20 mg/L) were inoculated with an overnight culture of *S. aureus* RN4220 pEPSA5-AS-*yycHI.* Expression of antisense RNA was induced with 50 mM xylose, a second culture without xylose served as control. The cultures were incubated at 25 °C for 30 h and examined by light-microscopy after Gram staining. The cell diameters were measured using ImageJ (NIH, Bethesda, MD, US; https://imagej.nih.gov/ij/) and statistics were performed employing GraphPad Prism 5 (GraphPad Software Inc., La Jolla, SD, USA) using a two-tailed Student’s t-test.

### 2.5. WTA Purification and Quantification

Purification of WTA from *S. aureus* cells was performed as described previously with minor modifications [29]. Briefly, overnight cultures in TSB supplemented with 50 mM xylose of the *S. aureus* HG003 strains containing the plasmids pEPSA5 and pEPSA5-AS-*yycHI* were harvested, washed with WTA-buffer 1 (50 mM MES, pH 6.5), and boiled in WTA-buffer 2 (50 mM MES, 4% SDS, pH 6.5) for 1 h. The lysed cells were washed 2 times in WTA-buffer 2 followed by WTA-buffer 3 (50 mM MES, 2% NaCl, pH 6.5) and again WTA-buffer 1. Proteins were degraded using 20 µg/mL Proteinase K in WTA-buffer 4 (20 mM Tris-HCl, 0.5% SDS, pH 8) for 4 h at 37 °C. The suspension was washed with WTA-buffer 3, then three times with ultrapure water and WTA were extracted using 0.1 mM NaOH. Quantification of the inorganic phosphate content was performed exactly as for the phospholipid liposomes [30].

### 2.6. Peptidoglycan Purification and Muropeptide Analysis of the Antisense yycHI Strain

*S. aureus* HG003 strains carrying the empty pEPSA5 vector or the *yycHI* antisense plasmids were grown in LB supplemented with 50 mM xylose until exponential growth phase and harvested by centrifugation. Purification of peptidoglycan and UPLC analysis were performed as previously described [31] with modifications of the protocol as follows: Mutanolysin digestion was performed with 20 µL (1000 U/mL) of mutanolysin for 20 h at 37 °C with agitation in 1.5 mL reaction tubes. The UPLC injection volume was 50 µL and solvents were replaced by 100 mM NaH_2_PO_4_ at pH 2.5 with 5% methanol for solvent A and pH 2.8 with 30% methanol for solvent B, according to [32].

### 2.7. Zymogram Analysis of Autolysin Extracts

*S. aureus* strains NRS384 *walR*-FLAG and NRS384 *walR*-FLAG Δ*yycHI* were grown in 50 mL TSB and strains HG003 pEPSA5 and HG003 pEPSA5-AS-*yycHI* in TSB containing 50 mM D-xylose and 34 µg/mL chloramphenicol until an OD_600_ of 1. The cells were harvested by centrifugation (5800× *g*, 4 °C, 20 min), washed with ultrapure water, and resuspended in 200 µL extraction buffer (50 mM Tris-HCl, 3 M LiCl, pH 7). After incubation on ice for 30 min, the suspension was centrifuged (7300× *g*, 4 °C, 5 min) and the supernatant was run in an acrylamide gel containing pasteurized *Micrococcus luteus* cells. The gel was washed 3 times for 20 min with deionized water and incubated in zymogram buffer (50 mM Tris-HCl, 10 mM CaCl_2_, 10 mM MgCl_2_, 0.1% (*v*/*v*) Triton X-100, pH 7.5) for 12 h at 37 °C with agitation. For better visibility of the clear bands, the gel was stained with 0.1% methylene blue (*w*/*v*).

### 2.8. Overexpression and Purification of WalK, YycH, and YycI

A detailed protocol used for overexpression and purification has been described previously [33]. Briefly, *E. coli* strains containing the expression plasmids were incubated in LB broth supplemented with 100 mg/L ampicillin and 50 mg/L kanamycin at 37 °C in a shaking water bath until exponential growth phase. IPTG was added to a final concentration of 1 mM for induction of the expression of the desired protein. For expression of His_6_-tagged WalK, YycH, and YycI, *E. coli* C43(DE3) containing a constitutively expressed chaperone encoded on the pREP4*groESL*(MT) vector [34] and the plasmids pET22bΔpelB_*walK*, pET22bΔpelB_*yycH*, and pET22bΔpelB_*yycI* were incubated at 30 °C in a shaking water bath for 18 h. The cells were harvested by centrifugation and pellets were resuspended in lysis buffer 1 (50 mM NaH_2_PO_4_, 300 mM NaCl, 10 mM imidazole, 2 mM β-mercaptoethanol, 30% (*v*/*v*) glycerol, pH 8), treated with 200 µg/mL lysozyme and 25 U/mL Endonuclease (Benzonase^®^ Nuclease, Merck, Darmstadt, Germany) for 30 min and lysed by ultrasonication. Cell debris was removed by centrifugation, followed by additional ultracentrifugation of the lysate. The pellet was resuspended in lysis buffer 2 (50 mM NaH_2_PO_4_, 300 mM NaCl, 20 mM imidazole, 40 mM dodecyl-β-D-maltoside (DDM), 2 mM β-mercaptoethanol, 30% (*v*/*v*) glycerol, pH 8). After further ultracentrifugation the supernatant was loaded onto a gravity-flow column containing Ni-NTA affinity resin. The column was washed with wash buffer 1 (50 mM NaH_2_PO_4_, 300 mM NaCl, 20 mM imidazole, 4 mM DDM, 2 mM β-mercaptoethanol, 30% (*v*/*v*) glycerol, pH 8) and wash buffer 2 (50 mM NaH_2_PO_4_, 300 mM NaCl, 40 mM imidazole, 4 mM DDM, 2 mM β-mercaptoethanol, 30% (*v*/*v*) glycerol, pH 8) and the His_6_-tagged proteins were eluted with elution buffer (50 mM NaH_2_PO_4_, 300 mM NaCl, 300 mM imidazole, 4 mM DDM, 2 mM β-mercaptoethanol, 30% (*v*/*v*) glycerol, pH 8). The proteins were dialyzed (50 mM HEPES (N-2-Hydroxyethylpiperazine-N′-2-ethanesulfonic acid), 200 mM KCl, 50% (*v*/*v*) glycerol, pH 8) using Slide-A-Lyzer™ Dialysis Cassettes (Thermo Fisher Scientific), supplemented with glycerol to a final concentration of 50% and stored at –20 °C.

### 2.9. Reconstitution of Membrane Proteins into Phospholipid Liposomes

Proteoliposomes were created as previously described with modifications [34]. First, 3 mg of *E. coli* polar lipid extract (Avanti Polar Lipids, Alabaster, Alabama, USA) were suspended in 1 mL 100 mM phosphate buffer (pH 7.5) and extruded 19 times through a polycarbonate membrane (Whatman^®^, Nucleopore™ PC Track-Etched Membrane, 19 mm, 0.4 μm) using a mini extruder (Avanti Polar Lipids). The liposomes were titrated with Triton X-100 until saturation and 50 µmol of the WalK kinase as well as different ratios of YycH and YycI were added to the reaction. Triton X-100 was subsequently removed from the liposomes using an adsorbent (Bio-Beads SM-2, Bio-Rad). The liposomes were ultracentrifuged, resuspended in 1 mL phosphorylation buffer (50 mM HEPES, 500 mM KCl, 5 mM MgCl_2_, 0.5 mM dithiothreitol, 3.5% (*v*/*v*) glycerol, pH 8), and extruded again as described above. After another ultracentrifugation step, the liposomes were resuspended in 100 µL of phosphorylation buffer and inorganic phosphate content of every batch of liposomes was quantified as previously described [30]. The proteoliposomes were used in phosphorylation assays or stored at 4 °C.

### 2.10. Phosphorylation Assays with WalK in Phospholipid Liposomes

Phosphorylation assays of the liposomes containing the reconstituted proteins were performed as previously described [33]. Proteoliposomes were mixed with 1 µCi/µL [γ^32^P]-ATP in phosphorylation buffer (50 mM HEPES, 500 mM KCl, 25 mM MgCl_2_, 0.5 mM DTT, 3.5% (*v*/*v*) glycerol, pH 8) and incubated at 15 °C for 30 min. This time interval was chosen, because it was needed for a visible phosphorylation of the control and enabled the observation of inhibitory or activating effects. The reaction was stopped with 2x Laemmli SDS sample buffer and loaded onto a pre-cast SDS-PAGE gel (NuPAGE Bis Tris Gels, Thermo Fisher Scientific). Electrophoresis was performed in accordance to the manufacturer’s instructions. The radioactive bands were visualized using a storage phosphor screen (GE Healthcare, Solingen, Germany) with digitalization and quantification using the Storm Scanner System and ImageQuant TL software.

### 2.11. Phosphorylation Assays with Peptidoglycan Fragments, D-Alanine, and WTA in Detergent Micelles

For extraction of autolysins, *S. aureus* HG003 was grown in TSB at 37 °C until OD_600_ = 1.5 and cooled on ice immediately. Cells were harvested by centrifugation at 5800× *g* for 15 min at 4 °C and resuspended in extraction buffer (50 mM Tris-HCl, 3 M LiCl, pH 7). After 30 min incubation on ice, the suspension was centrifuged at 15,000× *g* and 4 °C for 5 min and the supernatant was used as an autolysin extract immediately. For lysis, WTA-free peptidoglycan from *S. aureus* HG003 was purified as described in the methods section for muropeptide analysis. Then, 30 µL of the peptidoglycan suspension were diluted in 1 mL PBS and 50 µL of the autolysin extract as well as 200 ng/mL lysostaphin were added. Lysostaphin was added to replace LytM, which was shown to be inactive in in vitro experiments [35]. PGN was also once digested with autolysin extract, which was concentrated by Vivaspin^®^ 3000 MWCO, without the addition of lysostaphin. Lysis was observed as the reduction of OD_600_ over time. After 120 min of lysis, the suspension was spun down and the supernatant was used for the phosphorylation assays with WalK and WalR. Phosphorylation assays with detergent-micelles were performed using the Phos-tag SDS-PAGE method for histidine kinases as previously described [33]. Prior to addition of ATP, the kinase was incubated for 10 min with 500 µM N-acetylglucosamine, 500 µM N-acetylmuramic acid, 500 µM of the peptidoglycan pentapeptide (Ala-d-γ-Glu-Lys-d-Ala-D-Ala), 1 mM d-alanine (all from Sigma), 1.5 to 6 µL of the lysed peptidoglycan, or 2 µL of purified WTA as described in the WTA extraction and quantification section. Phos-tag SDS-PAGE of the samples was conducted as described above. The Phos-tag gels were silver stained and the phosphorylation of WalR in presence of the supplement was compared to a sample without supplement.

## 3. Results

### 3.1. Inactivation of yycHI Expression Leads to Reduced Autolysis

To investigate the regulatory role of YycH and YycI in *S. aureus*, we created antisense knockdowns of the *yycH* and *yycI* genes in the laboratory methicillin-sensitive *S. aureus* strain HG003 using the xylose-inducible pEPSA5 vector [25,26]. The knockdown of YycH and YycI was confirmed by Western blots using specific anti-YycH and anti-YycI antibodies. The quantity of the YycH and YycI proteins was reduced by antisense RNA in *S. aureus* HG003 (Figure 2a). Inhibition of translation of single genes by antisense RNA also affects other genes in the same operon to some extent due to the polycistronic transcription in prokaryotes [36]. Thus, induction of *yycH* antisense RNA also reduced the abundance of YycI in the cell and vice versa (Figure 2a). To test whether WalR abundance was impacted in the antisense strains, the antisense plasmids were transformed into *S. aureus* NRS384 *walR*-FLAG and a Western blot of the cell lysates using an anti-FLAG antibody was performed which showed no difference in WalR abundance (Figure 2a). Because YycH abundance was reduced upon induction of the *yycI* antisense RNA and vice versa, we present only results of the *yycHI* double knockdown in comparison to the empty pEPSA5 vector control in the other experiments. Exponentially growing cells of these strains were harvested and the decrease of absorbance was observed after addition of 0.1% Triton X-100. After 180 min, the initial OD_600_ of the empty vector control cells decreased to approximately 30% of the initial, whereas the OD_600_ of the *yycHI* knockdown strain only reduced to 55% (Figure 2b). This means that decreasing levels of YycH and YycI impaired autolysis, indicating a lower abundance or activity of autolysins. This was confirmed by zymogram analysis of the *yycHI* knockdown strain. Autolysin extracts from exponential phase were run in an acrylamide gel containing *M. luteus* cells. The *yycHI* knockdown strain showed less autolytic activity in the zymogram in comparison to the empty vector control, which was particularly pronounced in the glucosaminidase domain of AtlA (Figure 2c). This was also the case for *S. aureus* NRS384 *walR*-FLAG Δ*yycHI* with chromosomal deletion of the *yycHI* genes in comparison to the respective strain without the *yycHI* deletion, *S. aureus* NRS384 *walR*-FLAG (Figure 2c). These strains were used for the analysis of WalR phosphorylation (see below). Light microscopy of antisense cultures showed that some cells were enlarged compared to the control. This phenomenon was strongest after 30 h of incubation (Figure S1).

Therefore, the YycH and YycI proteins influence the WalRK TCS in a positive manner. Alterations in autolysis behavior caused by chromosomal deletions of the *yycH* and *yycI* genes had already been reported [13]. We show here that the antisense knockdown strain has the same effect on production or activity of autolysins.

### 3.2. Reduction in YycH and YycI Levels Increases WTA Content of S. aureus Cell Walls

Autolysis during cell division is not only regulated by modulating autolysin expression or activity of the cell wall hydrolases, but also through mechanisms that coordinate the correct localization of these enzymes. WTA is involved in regulation of the spatio-temporal organization of the major staphylococcal autolysin AtlA via an exclusion strategy, hindering its binding to peptidoglycan outside the division septum during cell division [28]. We tested whether the WTA content of the cell walls is affected when the production of YycH and YycI is reduced. Downregulation of *yycH* and *yycI* in the *S. aureus* HG003 antisense *yycHI* strain led to a higher WTA content in the cell walls as determined by quantification of the inorganic phosphate content (Figure 2d). A higher WTA content would result in less AtlA being located at the cell division site and could help to explain the results of the autolysis assays.

### 3.3. Muropeptide Composition of the Cell Wall Is Not Affected by YycH and YycI

By reducing the abundance of YycH and YycI in *S. aureus* HG003 cells, a slower autolysis was observed. To test whether this is correlated to differences in muropeptide composition, cell walls from *S. aureus* HG003 containing the antisense plasmid pEPSA5-AS-*yycHI* and the empty vector were purified, digested with mutanolysin, and analyzed by UPLC. Exponentially growing cells of the YycHI antisense knockdown strain did not show any difference in their peptidoglycan patterns when compared to the empty vector strain (Figure 2e).

### 3.4. Both Accessory Proteins YycH and YycI Are Required for Full Activation of WalK Autophosphorylation in Phospholipid Liposomes

**Figure 3:**
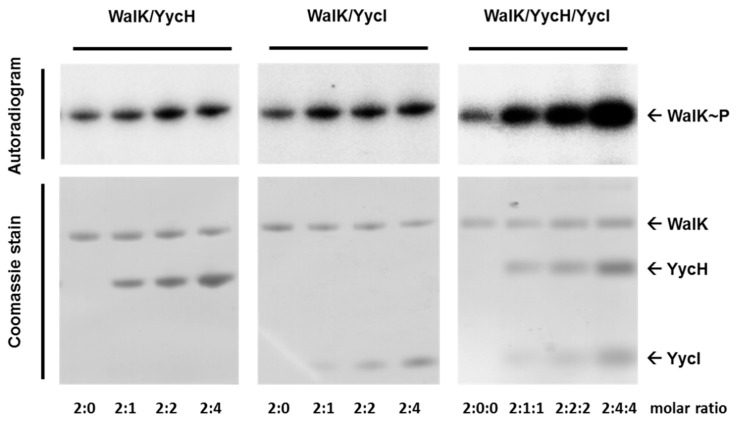
Activation of WalK autophosphorylation by YycH and YycI in phospholipid liposomes. WalK was reconstituted separately and with increasing amounts of YycH and/or YycI into phospholipid liposomes with molar ratios of 2:1, 2:2, and 2:4 for WalK(monomer)/YycH and WalK/YycI and 2:1:1, 2:2:2, and 2:4:4 for WalK/YycH/YycI. Autophosphorylation was started using [γ^32^P]-ATP and liposomes were loaded on a 10% SDS-PAGE gel. WalK with bound radiolabeled phosphate was visualized using a storage phosphor screen (top) and the gels were stained with Coomassie afterwards (bottom). Representative autoradiograms and Coomassie stained gels of three different experiments are shown. YycH and YycI increased the autophosphorylation of WalK, especially when both regulatory proteins were present in the liposomes.

In addition to the characterization of the antisense *yycHI* strain, we wanted to show a direct impact of YycH and YycI on the autophosphorylation activity of WalK in vitro. Combinations of WalK, YycH, and YycI were reconstituted into phospholipid liposomes and the kinase activity was tested using [γ^32^P]-ATP, followed by SDS-PAGE. After autoradiography, the gels were stained with Coomassie Brilliant Blue R250. By increasing the ratio of both YycH and YycI, in relation to the WalK kinase, an increasing autophosphorylation activity was observed. In comparison to the liposomes harboring only the kinase, the signal was much stronger with the highest amount of YycH and YycI and a molar ratio of 1:2:2 for the WalK monomer/YycH/YycI (Figure 3). Liposomes containing increasing amounts of either YycH or YycI also showed a slight increase in WalK activity, however, WalK was less activated than in the liposomes containing both regulatory proteins.

### 3.5. YycH and YycI Stimulate WalR Phosphorylation

**Figure 4:**
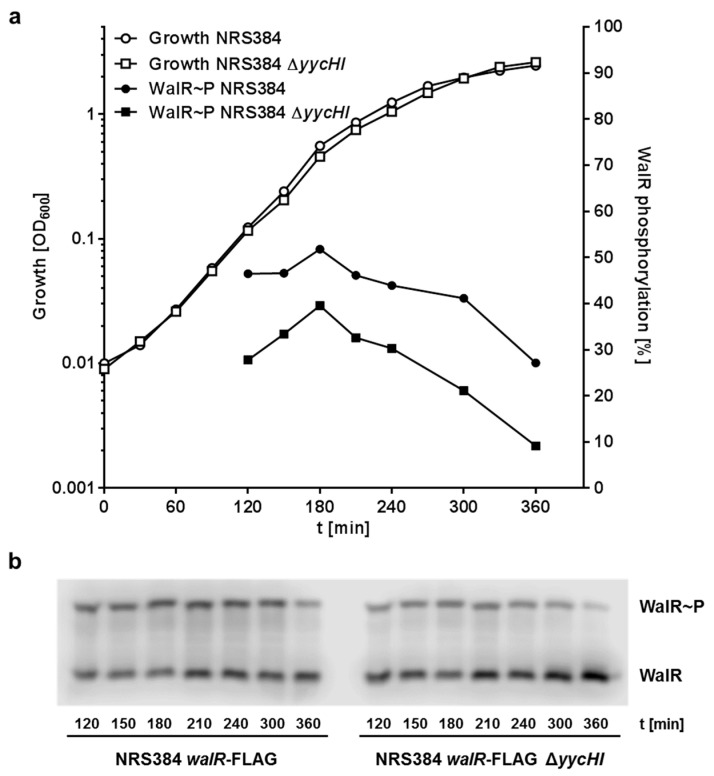
Deletion of *yycH* and *yycI* reduces WalR phosphorylation. (**a**) Growth and WalR phosphorylation of *S. aureus* NRS384 *walR*-FLAG and NRS384 *walR*-FLAG Δ*yycHI* as determined by OD_600_ measurement of the cultures and WalR-FLAG detection by quantification of the phosphorylated and non-phosphorylated bands of the Phos-Tag Western blots. (**b**) Phos-Tag Western blot of *S. aureus* NRS384 *walR*-FLAG and NRS384 *walR*-FLAG Δ*yycHI* at different time points. In all samples, the phosphorylation of WalR was higher in the wild-type strain than in the *yycHI* deletion mutant. The experiment was performed three times and the figure shows a representative result.

To test the influence of YycH and YycI on the phosphorylation state of the WalR response regulator in *S. aureus* cells, immunoblotting was performed using cell lysates of *S. aureus* NRS384 *walR*-FLAG with and without the deletion of *yycHI*. Due to the volume of culture necessary for sufficient protein, no samples could be taken below OD_600_ = 0.1. The level of WalR phosphorylation increased until mid-log phase and subsequently decreased as the cells transitioned into stationary phase (Figure 4). More WalR phosphorylation was observed in the wild-type than in the YycHI deficient strain throughout all stages of growth. This result indicates that YycH and YycI interact with WalK and support the phosphorylation of WalR and therefore are important players in the regulation of WalK activity, albeit not being essential for growth.

### 3.6. Cell Wall Fragments Did Not Affect WalRK Phosphorylation in In Vitro Phosphorylation Assays

We tested several cell wall components for their ability to alter the phosphorylation of WalR by WalK in vitro. N-acetylmuramic acid, N-acetylglucosamine, and the peptidoglycan pentapeptide (Ala-d-γ-Glu-Lys-d-Ala-d-Ala) are cell wall components that should be liberated after lysis of the peptidoglycan polymer by the glucosaminidase AtlA, transglycosylase IsaA, muramidase SceD, amidases AtlA and SsaA, the endopeptidase LytM, and other enzymes that are all regulated by the WalRK TCS [3] and that might interact with the extracellular PAS domain of WalK. In order to investigate a putative autolysis feedback mechanism, we added these components to in vitro phosphorylation assays with WalK and WalR and performed Phos-Tag SDS-PAGE. The phosphorylation of WalR by WalK was not impacted by any of the tested cell wall components (Figure S2a). We also tested D-alanine, which is available in the extracytoplasmic space after transpeptidation by penicillin binding proteins and which might be a signal indicating an ongoing cell wall biosynthesis. However, D-alanine did not have any effect on WalRK activity in the Phos-Tag SDS-PAGE assays (Figure S1a). Additionally, purified peptidoglycan was lysed with extracted autolysins from *S. aureus* with and without the addition of lysostaphin. The resulting cell wall fragments did not show any effect on phosphorylation of WalR either (Figure S2b). However, such an effect might be still mediated by a cell wall fragment from the digest that did not reach the threshold concentration in the extract used in our assays or was not produced, because of the in vitro inactivity of LytM (50). In conclusion, a putative autolysis feedback mechanism based on sensing of lysed cell wall polymers could not be observed in vitro. Peptidoglycan biosynthesis, WTA biosynthesis, and capsule biosynthesis are tightly connected because all three share a critical molecule, namely undecaprenyl-pyrophosphate. The availability of this lipid carrier needs to be coordinated for each of these pathways, hence, we thought of a regulation mechanism for WalRK activity via the availability of WTA. However, the addition of purified WTA from *S. aureus* cell walls to phosphorylation assays with WalK and WalR did not show any effect and it is more likely that a WTA precursor molecule might be a signal and not the fully developed WTA released from the cell wall (Figure S2a).

## 4. Discussion

Bacterial cell wall biosynthesis and division requires an interplay between biosynthesis of the new cell wall and peptidoglycan turnover to separate the dividing cells and to provide plasticity of the growing cell wall. Lysis is performed by a set of enzymes that are under the control of WalRK, making this two-component system essential for *S. aureus* [2]. The expression of these cell wall hydrolases needs to be carefully orchestrated to ensure a fully functioning peptidoglycan structure. Depletion of WalRK mediated autolysin expression leads to an abnormal cell wall that impairs the viability of *S. aureus* [37], whereas a higher activity of WalRK causes increased autolysis and a premature inflammatory response due to excessive peptidoglycan release, impairing pathogen survival in the host [37]. WalK has an extracytoplasmic and a cytoplasmic PAS domain, both being potential candidates for sensing ligands that control the phosphorylation activity. Crystallization of the cytoplasmic PAS domain revealed a Zinc atom binding site, which upon zinc-binding leads to a conformational change of WalK that inhibits WalRK phosphorylation [9]. Very recently it was shown that so far unidentified peptidoglycan degradation products modify the activity of *B. subtilis* WalK and might interact with the extracellular PAS domain [20]. In addition, there are different other mechanisms that control WalRK activity, mainly through interaction with other proteins. In *B. subtilis*, WalK interacts with DivIB, FtsL, Pbp2B, and FtsW, which are all cell division proteins that may well modulate WalK activity [22]. A cross-talk of the WalRK system and the Ser/Thr kinase PknB in *S. aureus* and PrkC in *B. subtilis* respectively, was discovered with the Ser/Thr kinases being able to phosphorylate the WalR response regulators at the T101 residue, whereas WalK phosphorylates D53 [38,39]. Recently, a protein that co-localizes with WalK in the division septum, SpdC, was identified as a positive regulator of WalRK activity in *S. aureus* [40].

In *B. subtilis*, YycH and YycI regulate WalRK activity in a negative manner, which is contrary to the activating role of these proteins in *S. aureus*, as shown by the results presented here and by other studies [13,22]. Previous in vitro experiments to demonstrate WalK activation by YycH and YycI failed due to a rather artificial test system. Triton X-100 was used to form detergent micelles, which probably bound to the hydrophobic trans-membrane domains of WalK, YycH, and YycI, thereby preventing an interaction of the proteins [34]. The trans-membrane domains of YycH and YycI are crucial for an interaction with WalK in *B. subtilis* and in *S. aureus* [13,21]. In this study, we used phospholipid liposomes, where full-length WalK, YycH, and YycI were reconstituted into a cell membrane-like environment and therefore could diffuse in the lipid bilayer. This gave the trans-membrane domains of the different proteins the possibility to interact within the bilayer and form a complex. For the first time, a direct activation of WalK by YycH and YycI could be demonstrated. Phosphorylation activity was increased, especially when both regulatory proteins had been reconstituted together with WalK in the liposomes in a ratio of 1:2:2 (WalK dimer:4YycH:4YycI). Compared to the complex that was postulated for *B. subtilis* (WalK dimer: 2 YycH: 2 YycI), an excess of the accessory proteins had to be used for full activation. However, the high concentrations may have been necessary to saturate complex formation in the liposomes, as the direction of the insertion into the membrane must be correct for all three proteins and therefore might be limiting in the liposome system.

An activating effect of YycH and YycI has been reported in a recent study with transcriptional profiling of deletion strains. Deletion of either *yycH* or *yycI* yielded almost identical transcriptomes, which differed from the wild-type and caused the transcription of the autolysins Sle1, AtlA, SsaA, and IsaA to be downregulated [13]. Vancomycin resistance increased in the *yycH*, *yycI*, and *yycHI* mutants with no significant differences between the strains, stating that both proteins are required for their regulating effect on WalRK. Furthermore, bacterial two-hybrid assays revealed repeatedly that WalK only interacts with a YycHI complex and not with the single proteins [13,20]. Our results support these findings, since full activation of WalK autophosphorylation was only achieved with both auxiliary proteins, YycH and YycI, in the liposomes.

By using Phos-Tag SDS-PAGE in combination with immunoblotting, we were able to visualize the phosphorylation state of the FLAG-tagged WalR response regulator in lysates of growing *S. aureus* cells. The absence of YycH and YycI in *S. aureus* NRS384 *walR*-FLAG Δ*yycHI* caused WalR to be less phosphorylated throughout all growth stages, which indicates the importance of YycH and YycI. WalR phosphorylation peaked at the end of the log phase, which is consistent with work conducted in *B. subtilis* [5,41] and in *S. aureus* [9].

We used an antisense RNA-based approach to knock down the production of YycH and YycI in *S. aureus* HG003. Immunoblotting of growing *S. aureus* cells with anti-YycH and anti-YycI antibodies confirmed the reduced abundance of the proteins in cell lysates. Due to the effect of the antisense RNA on the polycistronic transcript, knockdown of YycH or YycI alone also caused a double knockdown phenotype to some extent. It has been shown before that antisense RNA targeting single genes in a cistron also can affect the adjacent genes [36]. The expression of WalR in the antisense *yycHI* strain, however, was not affected. As WalRK is essential, the strain would not be able to grow under these conditions.

In the antisense *yycHI* strains, Triton X-100 induced autolysis was reduced and autolysin extracts showed less lysis of *M. luteus* cells in zymographic analysis. Some cells appeared enlarged and might have had problems to initiate cell septation. Similar defects (irregular septation and failure to initiate cell division) have been described in cells starved for WalRK [37]. Furthermore, the WTA content of the cell wall was increased. The reason for the increased WTA content and the role of WalRK during this effect remains unknown. In *B. subtilis*, a positive regulation of WTA biosynthesis genes by WalRK was reported [41]. Furthermore, peptidoglycan, WTA, and teichuronic acid biosyntheses are regulated by balancing of WalRK and PhoPR activity, the latter of which is dependent on phosphate availability in this organism [42,43]. In *S. aureus*, to the best of our knowledge, the regulation of WTA biosynthesis by WalRK has not been observed. However, the expression of WalRK controlled genes is increased together with WTA biosynthesis genes during the early phase of nasal colonization [8]. Interestingly, a higher concentration of WTA in the cell walls might affect autolysis, too, [36] and therefore contribute to the lower autolysis seen with the *yycHI* antisense strains. Further knockdown experiments employing a Δ*tagO* mutant of *S. aureus*, which is not able to produce teichoic acids [36], for knockdown of YycHI, might inform about the effect of teichoic acids on autolysis of the *yycHI* antisense strains. Alternatively, a comparison of the lysis of isolated cell walls of *yycHI* antisense strains with a control in the presence of externally added autolysins might help to decide whether the decreased autolysis is caused by the higher WTA content in the cell wall of the *yycHI* antisense strains or lower transcription of autolysins as expected in these strains [13] or a combination of both mechanisms.

Because the *yycHI* knockdown caused reduced autolysis, we analyzed if the cell wall muropeptide profile of this strain was affected. However, significant differences in the spectra of the muropeptide analysis could not be observed (Figure 2d). In previous studies, muropeptide analysis of a WalRK depleted strain showed only slight changes in comparison to the reference strain [3] and deletion of *atlA* showed no difference at all [44]. This suggests that peptidoglycan cross-linking is not affected by changes in autolysin regulation or WalK activity, but is more dependent on transglycosylation and transpeptidation by penicillin-binding proteins and WTA content of the cell wall.

Because WalK is localized in the septal region during cell division and, at least in *B. subtilis*, interacts with cell division proteins, the ultimate peptidoglycan precursor lipid II has been discussed as a signal for WalK [22,45]. WalR can be phosphorylated by PknB, a serine/threonine kinase that uses lipid II as a signal for coordination of autolysis and capsule biosynthesis, therefore additional sensing by WalK is doubtful [38,46]. Moreover, PknB needs all of its three extracytoplasmic PASTA domains (penicillin-binding protein and serine/threonine kinase associated) for effective lipid II binding. Crystallization of the *S. aureus* WalK extracytoplasmic PAS domain has shown a structure that is highly related to the histidine kinase extracytoplasmic PAS domains of *B. subtilis* KinD, *Klebsiella pneumoniae* CitA, *E. coli* DcuS, and *Sinorhizobium meliloti* DctB, whose signals are pyruvate, citrate, malate, and succinate, respectively [47,48,49,50]. These are all molecules that have a much lower molecular weight than lipid II and, in our hands, the membrane bound lipid II and the final soluble precursor that is attached to the lipid carrier undecaprenyl-pyrophosphate, UDP-*N*-acetylmuramic acid pentapeptide, did not impact WalK activity in vitro [51]. However, PknB was shown to react differently to each lipid II derivative with lipid II-Gly3 showing the highest activation of PknB autokinase activity. We focused on a putative autolysis feedback mechanism that is not dependent on peptidoglycan biosynthesis precursors but on products of lysed peptidoglycan during autolysis as putative signals for WalK as proposed for *B. subtilis* [20]. Despite not being able to find a molecule that alters the activity of WalK, we provide an in vitro system for signal analysis and additionally we are able to investigate the WalR phosphorylation state in lysates of *S. aureus* cells at different growth stages, which is an important tool for further signaling studies.

## 5. Conclusions

In conclusion, the regulation of WalK is complex and comprises different environmental signals that are transmitted by different domains of the molecule. Work in *B. subtilis* indicates that the extracellular PAS domain might provide feedback information about the activity of the autolysins [20]. Zinc binding to the intracellular PAS domain is special for staphylococci and enterococci and inhibition by zinc was mainly observed at the entry into stationary phase [9], indicating that zinc might act to decrease activity of WalK at this time point. The role of the zinc signaling for *S. aureus* is unclear, but zinc is essential for *S. aureus*, a co-factor of some autolytic amidases [52] and plays a role in bacterial infection and innate immunity [53]. In addition, YycH and YycI might function as spatial regulators via their membrane domains, since YycH was found only in the septum [7]. Therefore, YycH and YycI would be able to activate only those WalK molecules that are located in the division septum. Other septal proteins (PknB [38], SpdC [40]) may be involved in the regulation as well, making this histidine kinase one of the most enigmatic regulatory proteins.

## Figures and Tables

**Table 1:** Primers used in this study.

Primer	5′-3′ sequence 2	Restriction Site
AS-*yycH*_for	GAATCTAGAACGCATGTTTTTGCACCA	XbaI
AS-*yycH*_rev	ATGGAATTCTCGCTTCATCTTCGGACA	EcoRI
AS-*yycI*_for	GTTCTAGAGCGCGTATTTAAAGGTGCT	XbaI
AS-*yycI*_rev	TATGAATTCGCACCATCTGTGGGCTTA	EcoRI
AS-*yycHI*_for	CAGTCTAGACGTACCGCGTTGGTATGT	XbaI
AS-*yycHI*_rev	CTTGAATTCTGTGTGAGCGATTGACTTT	EcoRI

## Supplementary Materials

The following are available online at https://www.mdpi.com/2076-2607/8/6/870/s1, Figure S1: Bright field microscopy of S. aureus RN4220 pEPSA5-AS-yycHI with and without xylose after 30 h of incubation. Figure S2: Effect of lysed peptidoglycan fragments, d-alanine and WTA on the phosphorylation of WalRK in vitro.
