# Supplementary material for: YycH and YycI Regulate Expression of Staphylococcus aureus Autolysins by Activation of WalRK Phosphorylation
Source: Microorganisms. 2020 Jun 9;8(6):870. doi: 10.3390/microorganisms8060870 (PMC7355866; doi:10.3390/microorganisms8060870)
Supplement: Supplementary file 1 [file microorganisms-08-00870-s001.pdf]

Supplementary Materials: Figure S1 and Figure S2

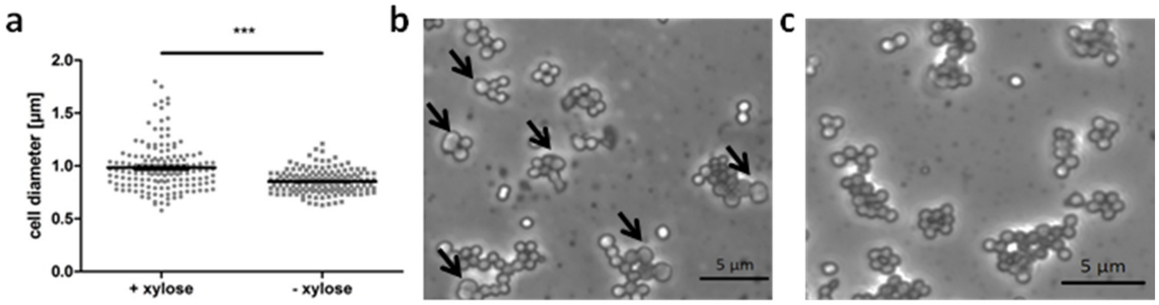

Figure S1: Bright field microscopy of *S. aureus* RN4220 pEPSA5-AS-*yycHI* with and without xylose after 30 h of incubation. (a) Measurement of cell diameters of 150 cells of *S. aureus* RN4220 pEPSA5-AS-*yycHI* in the presence and absence of xylose. The difference in cell wall diameter between both cultures was significant (p<0.0001). (b) Bright field microscopy of *S. aureus* RN4220 pEPSA5-AS-*yycHI* in the presence of xylose. Enlarged cells are marked with arrows. (c) *S. aureus* RN4220 pEPSA5-AS-*yycHI* in the absence of xylose serving as control.

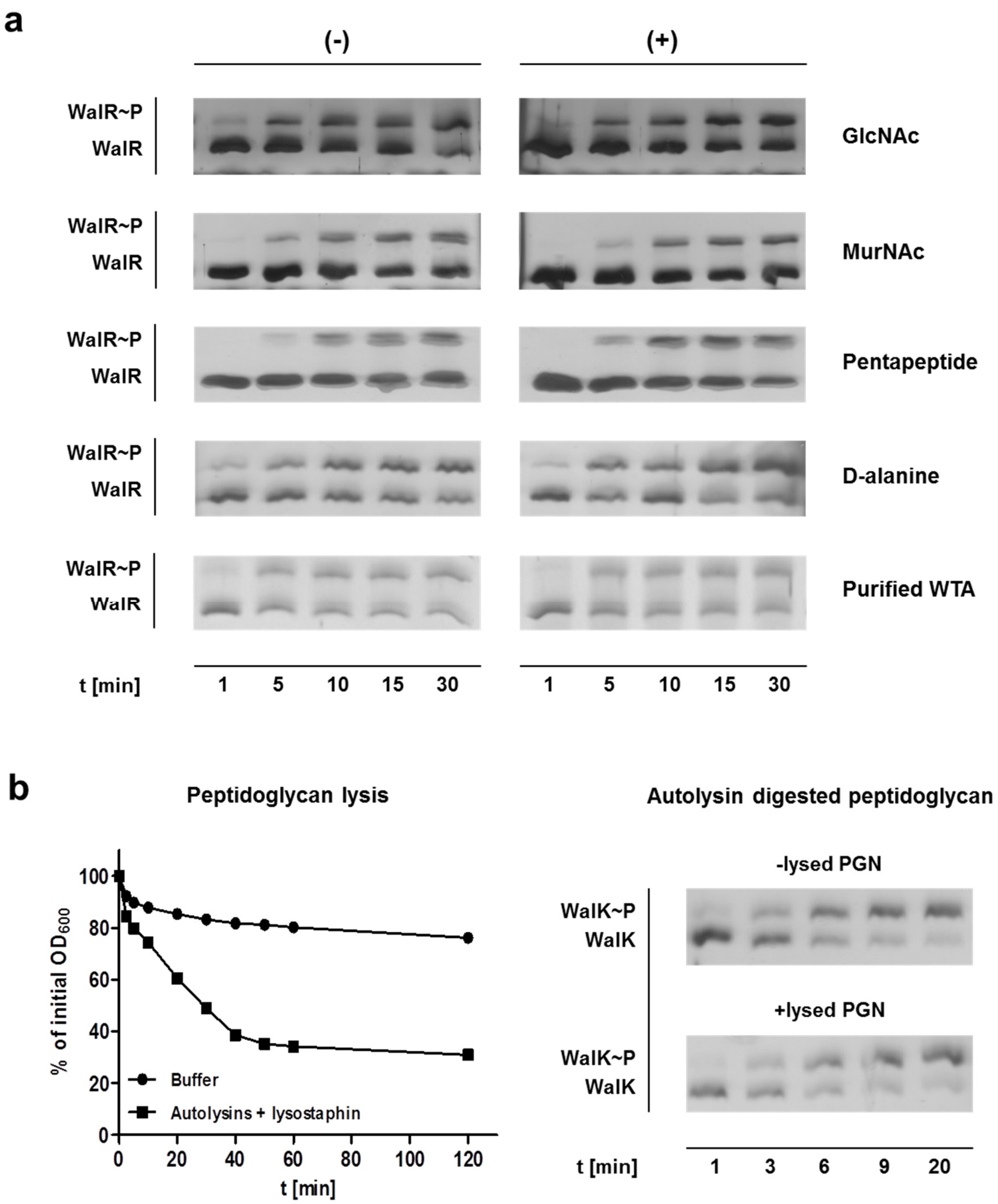

Figure S2. Effect of lysed peptidoglycan fragments, D-alanine and WTA on the phosphorylation of WalRK *in vitro*. (a) Purified recombinant WalK and WalR were incubated with 500 µM N-acetylglucosamine (GlcNAc), 500 µM N-acetylmuramic acid (MurNAc), 500 µM Ala-D-γ-Glu-Lys-D-Ala-D-Ala (pentapeptide), 1000 µM D-alanine or 2 µl of purified WTA or with the respective buffer. Phosphorylation assays were performed followed by Phos-tag SDS-PAGE. No differences in phosphorylation were observed by the addition of the compounds (+) in comparison to the respective buffer controls (-). (b) Purified peptidoglycan (PGN) was lysed by an autolysin extract together with lysostaphin as shown in the graph and added to a phosphorylation assay with the WalK kinase. This had no effect on phosphorylation.
